# Environmental transmission of *Toxoplasma gondii*: Oocysts in water, soil and food

**DOI:** 10.1016/j.fawpar.2019.e00049

**Published:** 2019-04-01

**Authors:** Karen Shapiro, Lillian Bahia-Oliveira, Brent Dixon, Aurélien Dumètre, Luz A. de Wit, Elizabeth VanWormer, Isabelle Villena

**Affiliations:** a Department of Pathology, Microbiology & Immunology, School of Veterinary Medicine, One Shields Ave, 4206 VM3A, University of California, Davis, CA 95616-5270, USA; b Laboratory of Immunoparasitology, Federal University of Rio de Janeiro, Macaé, RJ, Brazil; c Bureau of Microbial Hazards, Food Directorate, Health Canada, 251 Sir Frederick Banting Driveway, A.L. 2204E, Ottawa, ON K1A 0K9, Canada; d Aix Marseille Univ, IRD 257, AP-HM, SSA, VITROME, IHU-Méditerranée Infection, Marseille, France; e Department of Ecology and Evolutionary Biology, University of California Santa Cruz, 130 McAllister Way, Santa Cruz, CA 95050, USA; f School of Veterinary Medicine and Biomedical Sciences, School of Natural Resources, University of Nebraska-Lincoln, VBS 111, Lincoln, NE 68583, USA; g EA 7510, UFR Medicine, University Reims Champagne-Ardenne, National Reference Center on Toxoplasmosis, Hospital Maison Blanche, Reims, France

**Keywords:** *Toxoplasma gondii*, Oocyst, Transmission, Water, Soil, Food

## Abstract

*Toxoplasma gondii* is a zoonotic protozoan parasite that can cause morbidity and mortality in humans, domestic animals, and terrestrial and aquatic wildlife. The environmentally robust oocyst stage of *T. gondii* is fundamentally critical to the parasite's success, both in terms of its worldwide distribution as well as the extensive range of infected intermediate hosts. Despite the limited definitive host species (domestic and wild felids), infections have been reported on every continent, and in terrestrial as well as aquatic environments. The remarkable resistance of the oocyst wall enables dissemination of *T. gondii* through watersheds and ecosystems, and long-term persistence in diverse foods such as shellfish and fresh produce. Here, we review the key attributes of oocyst biophysical properties that confer their ability to disseminate and survive in the environment, as well as the epidemiological dynamics of oocyst sources including domestic and wild felids. This manuscript further provides a comprehensive review of the pathways by which *T. gondii* oocysts can infect animals and people through the environment, including in contaminated foods, water or soil. We conclude by identifying critical control points for reducing risk of exposure to oocysts as well as opportunities for future synergies and new directions for research aimed at reducing the burden of oocyst-borne toxoplasmosis in humans, domestic animals, and wildlife.

## Highlights

• Oocyst ingestion constitutes a significant proportion of *T. gondii* infections.
• Oocyst attributes facilitate *T. gondii* transmission in terrestrial and aquatic habitats.
• Oocysts are persistent and prevalent in water, soil and foods.
• Mitigating oocyst-borne infections requires transdisciplinary international efforts.

## 1 Introduction: the importance of oocysts in *Toxoplasma gondii* transmission

*Toxoplasma gondii* is an apicomplexan protozoan parasite that infects birds and mammals, including humans (Dubey and Beattie, 1988). Although infections are often asymptomatic, *T. gondii* can cause serious disease and death in humans and animals (Carme et al., 2002; Kreuder et al., 2003; Jones et al., 2012). The three primary transmission routes for *T. gondii* include vertical transmission from mother to fetus, ingestion of tissue cysts in infected animal tissues, and ingestion of oocysts from contaminated water, soil, or foods (Tenter et al., 2000). Since the parasite's discovery in 1908 (Ferguson, 2009), environmental transmission has arguably been the least studied route, likely due to the logistical constraints of safely producing large numbers of oocysts under laboratory conditions (Fritz et al., 2012a) and lack of standardized methods for detection of oocysts in complex environmental matrices (Dumetre and Darde, 2003).

Oocysts, the environmentally robust stage of *T. gondii*, play an important role in the epidemiology of this zoonotic parasite. Attributes of oocysts' biology may further explain the global distribution of *T. gondii* and the means by which it has evolved to be one of the most prevalent infectious agents of animals and humans (Dubey, 2004; Dumetre et al., 2012). While oocysts are exclusively deposited on land due to definitive hosts being solely terrestrial animals, the prevalent nature of infections observed in aquatic animals demonstrates a significant role for waterborne transmission. High prevalences of *T. gondii* exposure in marine species (up to 100% in some populations (Dubey et al., 2003)) further suggests that oocyst transport to, and accumulation in, nearshore or open ocean habitats is possible and epidemiologically significant (Miller et al., 2018).

Reports documenting the presence of *T. gondii* in diverse environmental matrices, including water, soil, vegetables and seafood have been increasing. New methods that can discriminate the route of *T. gondii* acquisition have demonstrated that, in some populations, a significant proportion of infections are caused by oocyst ingestion (Hill et al., 2011). Despite these findings, mainstream thinking in exposure mitigation for both humans and animals often neglects a comprehensive understanding and management of factors that are important to reducing the risk of exposure to oocysts. Therefore, this review aims to (i) summarize critical aspects of oocyst biology, environmental resistance, and felid dynamics of oocyst shedding; (ii) review the importance of oocyst-borne infections in human and animal populations; (iii) synthesize current knowledge on oocyst contamination of water, soil, fresh produce and seafoods; and (iv) identify critical gaps in current knowledge where further research and collaborative efforts should be directed to reduce *T. gondii* infections in animals and humans.

## 2 Felid dynamics of oocyst shedding

Although *Toxoplasma gondii* infects diverse species of warm-blooded animals, domestic cats (*Felis catus*) and wild felids are the only known definitive hosts capable of shedding environmentally hardy oocysts in their feces (Dubey et al., 1970; Hutchison et al., 1969; (Jewell et al., 1972; Miller et al., 1972). Domestic cats exist in close association with most human settlements throughout the world (Liberg et al., 2000), and both pet cats and free-ranging stray or feral domestic cats contribute to environmental oocyst burden. One or more of the 36 species of wild felids inhabit every continent except Australia and Antarctica (Macdonald and Loveridge, 2010), with multi-species assemblages present in many areas. *Toxoplasma* oocyst shedding has been identified microscopically and molecularly confirmed in free-ranging individuals from diverse wild felid species in North, Central and South America, and Asia (as reviewed in (Dubey, 2009; VanWormer et al., 2013a)), and likely occurs in all wild felids.

Oocyst contributions to the environment begin with infection of a felid host. Domestic cats and wild felids can be infected with *T. gondii* by consuming the tissues of an infected intermediate host (bradyzoite cysts), ingesting oocysts, or through congenital transmission (Dubey and Jones, 2008). The majority of felid infections are thought to be acquired through infected prey (Dubey and Jones, 2008), and the prevalence of oocyst shedding was higher in cats experimentally-infected with bradyzoites compared to those infected with tachyzoites or oocysts (Dubey, 2009) (Dubey and Frenkel, 1976). Following parasite ingestion, asexual and sexual reproduction of *T. gondii* occur in the felid's small intestine (Dubey, 2009). Genetic recombination and re-assortment during sexual reproduction has the potential to produce new strains of *T. gondii* (Elmore et al., 2010). Oocysts produced through sexual reproduction are shed in felid feces and sporulate one to five days later in the environment, becoming infective to other intermediate or definitive hosts (Elmore et al., 2010). When infected by ingesting tissue cysts, the natural exposure route for felids consuming infected prey, domestic cats can shed up to one billion oocysts typically over a 1–2 week period (Dubey and Frenkel, 1972; Dubey, 1995; Fritz et al., 2012a). The number of oocysts shed by experimentally-infected wild felids has not been quantified. However, a naturally-infected, free-ranging mountain lion in Canada shed quantities of oocysts similar to those shed by naturally-infected domestic cats (1.25 × 10^6^ per gram of feces; (Aramini et al., 1998)). Young cats may shed higher numbers of oocysts, and higher prevalence of oocyst shedding was observed in pet and feral kittens relative to adult domestic cats (Ruiz and Frenkel, 1980; Dubey and Beattie, 1988) (Dubey and Beattie, 1988; Ruiz and Frenkel, 1980). However, oocyst shedding was reported in naturally-infected adult domestic cats (1–18 years of age) in Europe and the United States (Schares et al., 2008; Herrmann et al., 2010; Berger-Schoch et al., 2011; VanWormer et al., 2013b).

The strain of *T. gondii* infecting a domestic or wild felid may impact both the number of oocysts shed by an individual cat as well as the prevalence of oocyst shedding. Domestic cats experimentally infected with certain strains of *T. gondii* shed higher numbers of oocysts per cat, but limited parasite genotypes were tested (Dubey, 1995). Experimental infection of domestic cats and bobcats (*Lynx rufus*) with an identical strain of *T. gondii* isolated from domestic sheep resulted in lower oocyst shedding prevalence in the wild felids (Dubey et al., 1970; Miller et al., 1972). However, wild felid shedding prevalence may increase with exposure to wild or atypical strains of *T. gondii.* Asian leopard cats (*Prionailurus bengalensis*) experimentally infected with *T. gondii* strains isolated from domestic or wild animals only shed oocysts when exposed to the wild strain (Miller et al., 1972). In naturally-infected pet and feral domestic cat populations, reported shedding prevalence for molecularly or bioassay confirmed *T. gondii* oocysts ranged from 0 to 20%, with higher levels detected in feral cats when geographically overlapping populations of pet and feral cats were compared (Herrmann et al., 2010; Jones and Dubey, 2010; Berger-Schoch et al., 2011; Lilly and Wortham, 2013; VanWormer et al., 2013b; Mancianti et al., 2015; Veronesi et al., 2017; Nabi et al., 2018). The reported prevalence of cats shedding oocysts in naturally-infected, free-ranging wild felid populations varied widely (0–37%; (Jokelainen et al., 2013; Simon et al., 2013a; VanWormer et al., 2013b). In a field study on the west coast of the USA that compared oocyst shedding among sympatric wild and domestic felids, smaller wild felids (bobcats) and feral domestic cats feeding primarily on wild prey had higher levels of oocyst shedding than larger wild felids (mountain lions; *Puma concolor*) or feral domestic cats being fed by humans (VanWormer et al., 2013b). Reported levels of *T. gondii*-like and molecularly confirmed oocyst shedding also differed among wild felids in other areas of the USA, with higher prevalences observed in bobcats than mountain lions (Marchiondo et al., 1976).

Domestic and wild felid contributions to environmental oocyst load may also be impacted by the frequency of oocyst shedding. While many studies focus on young cats and the period of shedding following an animal's initial infection, experimental and field evidence supports the potential for repeated episodes of oocyst shedding over the course of a felid's life. In experimental settings, chronically infected domestic cats re-shed oocysts following glucocorticoid-induced immunosuppression, co-infection with another common feline parasite, *Cystoisospora felis*, and when infected with a new strain of *T. gondii* (Chessum, 1972; Dubey and Frenkel, 1974; Dubey, 1976; Zulpo et al., 2018). Repeat shedding occurred experimentally in both malnourished and well-nourished domestic cats (Ruiz and Frenkel, 1980). Captive wild felids naturally exposed to *T. gondii* in raw meat also repeatedly shed oocysts (Lukesova and Literak, 1998). Diet, which can vary drastically among felid species as well as within populations of a single species living in different environments (Hass, 2009; Plantinga et al., 2011), plays an important role in potential repeat shedding in free-ranging felids by influencing immune status along with exposure to *C. felis* and novel strains of *T. gondii.* Mixed infections with more than one strain of *T. gondii* have been identified in naturally-infected domestic and wild felids (VanWormer et al., 2014; Verma et al., 2017; Valenzuela-Moreno et al., 2019). Domestic cats fed by humans or scavenging from foods discarded by people as well as wild felids that consume fewer, larger prey animals or prey species with lower prevalence of *T. gondii* infection may have lower risk of repeat shedding and lower observed infection and oocyst shedding prevalences (Afonso et al., 2007; VanWormer et al., 2013b). It is currently unknown what proportion of naturally-infected, free-ranging domestic and wild felid populations may be re-shedding oocysts at a given time. Longitudinal studies are needed to enhance our understanding of repeat shedding in these groups. Oocyst shedding has long been associated with young cats, but due to the possibility of re-shedding, all cats should be considered in the dissemination of oocysts.

While both domestic and wild felids shed *T. gondii* oocysts, the number of felids present in a given area, prevalence of oocyst shedding, and numbers of oocysts shed impact their relative contributions to environmental oocyst load. Multiple factors, including human presence, land use, habitat, and prey availability, shape the numbers and species of felids present in diverse environments. Due to these diverse influences on felid presence and *T. gondii* oocyst shedding, field studies are critical to evaluate the relative contributions of domestic and wild felids to environmental *T. gondii* oocyst burden on local, regional, and global scales. Molecular epidemiology studies of parasite genotypes in geographically overlapping felids, intermediate and paratenic hosts illustrated that both domestic and wild felids can play a role in archetypal and atypical *T. gondii* infections (Miller et al., 2008; VanWormer et al., 2014; Shapiro et al., 2015). Statistical and geographic information system (GIS)-based modeling approaches have been used to assess relative oocyst contributions of domestic and wild felids in landscapes where multiple felid species exist, illustrating that both groups contribute to environmental *T. gondii* contamination (VanWormer et al., 2016). Interestingly, higher levels of *T. gondii* infection in populations of marine hosts were associated with proximity to areas with greater human presence where oocyst contributions were estimated to be higher for domestic cats than wild felids (VanWormer et al., 2016; Burgess et al., 2018). Infection in terrestrial and marine intermediate hosts, as well as paratenic hosts, in areas like Hawaii and New Zealand that lack wild felids underscore the importance of domestic cat contributions to environmental oocyst load (Roe et al., 2013; Barbieri et al., 2016; Coupe et al., 2018).

## 3 Structural, molecular, and biophysical attributes of oocysts

**Fig. 1:**
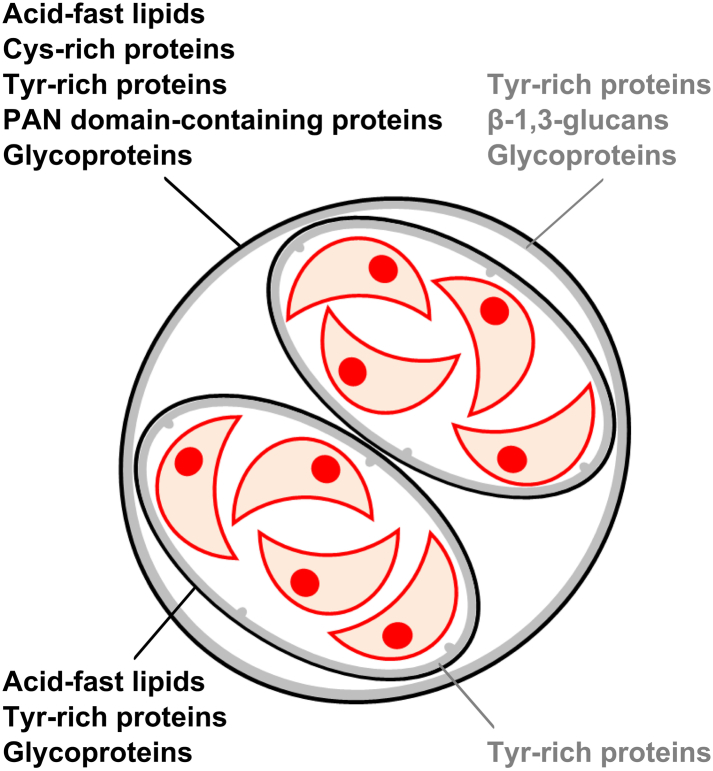
Structure of a sporulated *Toxoplasma gondii* oocyst and molecular composition of the walls enclosing the sporozoites (red crescent structures). (For interpretation of the references to colour in this figure legend, the reader is referred to the web version of this article.)

Excreted oocysts become infective following sporulation. This process results in the formation of two sporocysts each with four sporozoites (Figure 1). It is usually completed within 7 days at 20–25 °C under appropriate aerobic and humidity conditions, however it can be delayed at lower temperatures (4–11 °C) (Dubey et al., 1970), and even abrogated following constant freezing at −21 °C for 1 day or − 6 °C for 7 days (Frenkel and Dubey, 1973). Sporulated oocysts are considered to be more resistant to environmental insults than non-sporulated oocysts. This increase in resistance may be due to the additional presence of the sporocyst wall and the putative capacity of sporozoites to protect themselves against temperature variations, desiccation and ultraviolet radiation (Fritz et al., 2012b).

The structural, molecular, and biophysical properties of the oocyst and sporocyst walls play a key role in the environmental transmission of the parasite. These properties appear very conserved across the different parasite genotypes (Dumetre and Darde, 2007; Fritz et al., 2012b; Possenti et al., 2013). Both walls are bilayered and mainly proteinaceous (Mai et al., 2009). The outer oocyst wall layer (20 nm thick) contains cysteine-rich proteins of the oocyst wall protein (OWP) family and tyrosine-rich proteins (Possenti et al., 2010; Fritz et al., 2012b), which are prone to form robust polymeric structures through disulphide (Possenti et al., 2010) or dityrosine crosslinking (Fritz et al., 2012b), respectively. The latter are believed to be responsible for the natural blue fluorescence of oocyst and sporocyst walls. The additional presence of PAN domain-containing proteins could contribute to the stabilization of the architecture of the outer oocyst wall layer through disulfide bridging. Acid-fast lipids coat the oocyst surface and render the oocyst wall almost impermeable to water soluble molecules (Bushkin et al., 2013). The inner oocyst wall layer (30–70 nm thick) contains crosslinked Tyr-rich proteins and fibrils of β-1,3-glucans (Bushkin et al., 2012; Fritz et al., 2012b), which have a structural role. Several glycoproteins have been identified in both oocyst wall layers by using lectins (Samuelson et al., 2013; Harito et al., 2016), however their role is uncertain. The sporocyst wall appears to serve as a second level of protection. The outer sporocyst wall layer (15–20 nm thick) resembles the outer oocyst wall in structure and molecular composition except that it appears to lack OWP proteins. The inner sporocyst wall layer (40–50 nm) is made of four curved plates joined together by thick sutures. The unique structure of the inner sporocyst wall layer would provide additional mechanical resistance. Given the autofluorescence of both sporocyst wall layers, Tyr-rich proteins appear as the most abundant molecules in the sporocyst wall whereas β-1,3-glucans are absent (Freppel et al., 2019).

Due to their polymeric nature, the oocyst walls are resistant to mechanical perturbations (Dumetre et al., 2013) and almost hermetic to chemical inactivation agents, in particular strong acids, detergents, and chlorinated disinfectants (Jones and Dubey, 2010). For instance, diluted household bleach solutions can destroy the outer oocyst wall but do not significantly alter the structure, mechanics, and permeability of the inner oocyst wall, the sporocyst wall, nor the sporozoite infectivity (Dumetre et al., 2013). Oocyst walls similarly confer resistance to chlorine dioxide and chloramine at concentrations used in the water industry. In this regard, oocysts can pose health hazards in areas where people drink unfiltered chlorinated tap water (Jones and Dubey, 2010). Oocyst tolerance to other environmental stressors including salinity and enzymatic digestion, could also rely on the robust nature, molecular content and lack of permeability of the oocyst walls.

In addition to providing protection, the oocyst wall can mediate the retention or transport of oocysts in soils and waters in conjunction with environmental factors (Dumetre et al., 2012). The oocyst surface is hydrophilic, faintly adhesive and negatively charged in low-ionic strength solutions, suggesting that oocysts can mobilize from soils following heavy rainfalls and disperse in freshwaters (Dumetre et al., 2012, Dumetre et al., 2013; Shapiro et al., 2009). In contrast, oocyst surface charge approaches neutral in high-ionic strength solutions mimicking estuarine or marine waters (Shapiro et al., 2009), which can enhance oocyst interactions with marine biofilm and algae growing in coastal areas (Shapiro et al., 2014). This process results in oocyst incorporation into marine food webs, with exposure implications to higher tropic level animals as well as people. The oocyst wall properties, therefore, play a key role in the transmission dynamics of *T. gondii* across landscapes, as well as from land to aquatic habitats, facilitating exposure to numerous host species living in different biotopes worldwide (Dumetre et al., 2012).

The potential effect of *T. gondii* genotype on oocyst transport and persistence patterns in the environment is relatively unexplored. Some studies have demonstrated similar oocyst wall molecules via proteomic analyses performed on oocysts derived from genotypes II and III (Fritz et al., 2012a, Fritz et al., 2012b and Possenti et al. (2013), respectively); however, a more recent investigation by Zhou et al. (2017) identified differing expression of proteins between oocysts derived from a virulent phenotype (ToxoDB#9) vs those belonging to the less virulent Type II genotype. Additional investigations are warranted to evaluate if parasite genotype is associated with transport behavior or resistance to variable environmental stressors.

## 4 Importance of oocyst-borne infections

### 4.1 Oocyst-borne infections in humans

The epidemiologic importance of whether human toxoplasmosis is transmitted by oocysts or tissue cysts was debated even before the life cycle was determined in the 1970s (Jackson and Hutchison, 1989). The early observation in the 1950s that vegetarians and non-vegetarians had similar prevalence rates (Jacobs, 1957; Rawal, 1959) suggested that carnivorism could not be the only source of infection. Presently, the relative importance of oocyst vs tissue cyst ingestion for *Toxoplasma gondii* transmission in humans remains unknown for the majority of endemically infected populations.

Transmission patterns of *T. gondii* oocysts to people have been mostly characterized in toxoplasmosis outbreaks (Teutsch et al., 1979; Benenson et al., 1982; Coutinho et al., 1982; Bowie et al., 1997; de Moura et al., 2006; Ekman et al., 2012). Specifically, Brazil has experienced several oocyst-borne outbreaks, with water or produce implicated as the common source of exposure (Ferreira et al., 2018). Several factors that contribute to oocyst transmission patterns in Brazil are likely representative of other epidemic and/or endemic *T. gondii* regions, including precarious infrastructure for water and sewage treatment, large segments of the population that are poor and underserved, and inadequate access to healthcare. Endemic regions tend to include low-middle income countries, with detrimental consequences particularly evident in poor populations due to high rates of congenital and acquired toxoplasmosis (Bahia-Oliveira et al., 2017; El Bissati et al., 2018). The importance of *T. gondii* oocyst transmission in Brazil has been evaluated for both urban and peri-urban regions. Peri-urban regions are prevalent in this country and can be defined as the landscape interface between town and country. Specific factors that contribute to oocyst-borne infections in these regions include: 1) the high levels of environmental contamination with oocysts; 2) presence of diverse *T. gondii* genotypes and rich feline biodiversity in peri-urban, rural and forested areas; and 3) the lack of control of stray domestic cats living in urban and peri-urban areas. Combined, these features imply that exposure to oocysts likely contributes to the high prevalence of *T. gondii* infection in many populations in Brazil. The presence of diverse and atypical *T. gondii* strains in this region is also likely to contribute to acute disease outbreaks, as atypical genotypes were characterized as more virulent in several studies (Darde et al., 1998; Carme et al., 2002; Carme et al., 2009). Additional information on the role of parasite genotype and disease outcome is addressed in a separate manuscript in this Special Issue (Galal et al., 2019 FAWPAR, Present Special Issue on Toxoplasmosis & One Health).

**Fig. 2:**
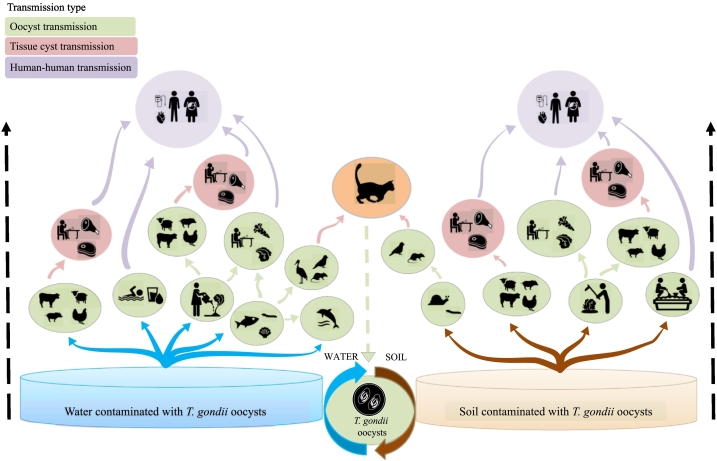
A *Toxoplasma gondii* oocyst transmission ‘tree’. The flux of environmental transport and infection with *T. gondii* starts with oocysts shed in cat feces that contaminate soil and/or water, and are subsequently transmitted to hosts (intermediate, paratenic and definitive). Green ovals and arrows represent different sources/scenarios of contamination/infections caused by ingestion of oocysts; pink ovals and arrows represent scenarios of transmission via bradyzoites (tissue cysts); and purple ovals and arrows represent human-to-human transmission caused by vertical (congenital), transfusional, or organ transplantation infections. Water is represented in blue with blue arrows depicting infection or contamination transmitted directly from water sources to hosts. Soil is represented in brown with brown arrows depicting infection or contamination transmitted directly from sources of soil to hosts. (For interpretation of the references to colour in this figure legend, the reader is referred to the web version of this article.)

Adapted from Bahia-Oliveira et al. (2017).

Ferreira et al. (2018) reported that from 25 outbreaks recorded in Brazil over the past 50 years, 56% (14/25) occurred between 2010 and 2018. Seventy two percent (18/25) had ingestion of oocysts in food, soil or water as the main risk factors; 24% (6/25) were associated with ingestion of tissue cysts from undercooked or raw meat; and 4% (1/25) were associated with tachyzoites ingested through unpasteurized milk. The low socioeconomic and educational circumstances in Brazil were identified as additional important risk factors for *T. gondii* infections in humans, and were associated with ingestion of oocysts in untreated water, consuming contaminated vegetables, or via contact with soil (Amendoeira et al., 2003; Bahia-Oliveira et al., 2003; Spalding et al., 2005; Cavalcante et al., 2006; Heukelbach et al., 2007; Boia et al., 2008; Sroka et al., 2010; Dattoli et al., 2011; Dias et al., 2011; Carellos et al., 2014). In terms of probability, exposure to oocysts in water or other matrices has been proposed to comprise a major risk for human infection both in developed and developing countries, largely due to the oocysts' long persistence in the environment and the diversity of paratenic hosts that can be ingested as food (e.g., shellfish, as represented in Fig. 2 (Bahia-Oliveira et al., 2017)).

An important methodological advancement for discriminating oocyst-borne infections has recently aided epidemiological investigations that aim to clarify the importance of different routes (oocysts vs. tissue cysts) of *T. gondii* transmission in people. These serological assays utilize a new generation of recombinant protein antigens that have been incorporated in ELISA systems and can detect immunoglobulins that recognize the sporozoite-specific embryogenesis-related protein (TgERP) (Hill et al., 2011), or the CCp5A, a recombinant protein derived from *T. gondii* sporozoites (Santana et al., 2015). When used together, both antigens, can further assist with establishing time since infection. Namely, antibodies against TgERP remain detectable in individuals' sera for 8 months after exposure, while antibodies against CCp5A are only detectable within 2 months of infection. In the USA and Chile, serological data regarding *T. gondii* sporozoite antigens indicate that oocysts were responsible for 78% and 45% of acute infections in pregnant woman, respectively (Boyer et al., 2011; Hill et al., 2011). The high rate of oocyst-borne infections in the USA is especially intriguing given that the overall seroprevalence for *T. gondii* has been declining in this country since the 1990s. The reduced burden of *T. gondii* infections in the USA has been largely attributed to improvements in biosecurity of farm animal management and meat preparation (e.g., thorough cooking), as well as an increase in hygiene-related safety guidelines (hand washing after gardening, safe handling of cat feces, etc.) (Jones et al., 2014). A similar pattern of declining *T. gondii* seroprevalence and associated explanatory variables has been observed in Europe (Pappas et al., 2009; Nogareda et al., 2014). However, increasing awareness and consumer demands for animal welfare may impact future patterns of toxoplasmosis in Europe as animals raised in free-ranging settings are more likely to be infected (Kijlstra and Jongert, 2009).

In Brazil, two studies investigated antibodies against TgERP in sera and saliva of persons exposed to drinking water contaminated with *T. gondii* oocysts and demonstrated that IgG was positive in 50% of individuals older than 20 years (Vieira et al., 2015), and that salivary IgA was detectable in 60% of patients 15 to 21 years of age (Mangiavacchi et al., 2016). Both studies raise important questions regarding the possibility of continuous reinfection in patients living in endemic settings under high pressure of environmental contamination with *T. gondii* oocysts. At the time of this review preparation, the largest *T. gondii* outbreak in Brazil had started in February 2018, and was still ongoing more one year after the initial cases were detected, with >700 serologically and clinically confirmed cases of acute toxoplasmosis. When applying the sporozoite antigen CCp5A-based serological assay in 36 of these confirmed cases, 78% (28/36) were positive, supporting the epidemiological finding implicating drinking water from faucets and eating vegetables washed with water from faucets as the main risk factors associated with *T. gondii* infection during this outbreak (Menegolla et al., 2018).

In terms of the relative epidemiologic importance of oocysts versus tissue cysts in the global *T. gondii* burden of infections, the new generation of recombinant sporozoite-specific antigens could serve as a valuable tool complementing epidemiological studies where often a single serological sample is available to estimate parameters such as prevalence (using conventional serological tests), mode of infection (oocysts versus tissue cysts) and the incidence (temporal rate of infection) in a given population. Additional studies validating the use of sporozoite-specific seroassays are needed to better understand the epidemiological value of these assays in discriminating the dynamics and route of *T. gondii* transmission in other geographical regions. These methodologies will thus facilitate logistics and cost efficiency of field investigations, as well as aid with determining the most appropriate local prevention measures against exposure to *T. gondii*, especially for pregnant women and immunocompromised patients.

### 4.2 Oocyst-borne infections in animals

The importance of oocysts as the primary infective stage responsible for transmission of *Toxoplasma gondii* to intermediate hosts such as rodents or birds, as well as herbivorous meat-producing animals, has been recognized since the life cycle of the parasite was initially described in 1969 (Jacobs et al., 1960; Hutchison et al., 1969; Dubey et al., 1970). In domestic farm animals, exposure to *T. gondii* is more likely if cats are present on farm premises and when animals are allowed greater access to free-roaming pastures (Waltnertoews et al., 1991; Jones and Dubey, 2012; Guo et al., 2015). In fact, free-roaming chickens have been used as an indicator of environmental oocyst contamination due to their feeding behavior in close proximity to contaminated soil (More et al., 2012). Of note are several reports demonstrating that husbandry operations with outdoor access result in higher likelihood of *T. gondii* infections in pigs (van der Giessen et al., 2007; Dubey et al., 2012) and chickens (Dubey, 2010) as compared with conventional operations, most likely resulting from greater access to natural outdoor habitats and free-ranging management strategies that lead to greater likelihood of exposure to contaminated soil or water. Higher burden of *T. gondii*-infected livestock is relevant for human public health not only for meat-producing animals, but also livestock producing dairy products. Several papers have documented the presence of *T. gondii* DNA (likely tachyzoites) in milk from goats and sheep (Mancianti et al., 2013; de Santana Rocha et al., 2015; Luptakova et al., 2015; Saad et al., 2018), and toxoplasmosis outbreak in people have been linked to consumption of unpasteurized milk in Brazil and the US (Sacks et al., 1982; Ferreira et al., 2018). As consumer demands are rising for improved animal welfare and more natural farming conditions, there is a potential for increasing proportions of *T. gondii*-infected meat- and/or dairy-producing livestock, with cascading public health implications.

In wildlife, oocyst-borne *T. gondii* infections have been proposed to occur in numerous species including freshwater-dwelling mammals (Ribas et al., 2018), ungulates (Dubey et al., 2017), and birds (Frenkel et al., 1995). In the past decade, several studies have specifically focused on the curious finding of high prevalence of *T. gondii* in marine mammals (Fayer et al., 2004). A thorough review on the global prevalence and distribution of *T. gondii* infections in marine mammals was recently compiled by Miller et al. (2018). Clinical toxoplasmosis has been documented in cetaceans, phocids, otariids, walruses, sirenians and sea otters. Serological exposure has been further documented in species sampled from coastal habitats to the deep sea, and across all continents including arctic locales and Antarctica where felids are completely absent.

The mechanism of land-to-sea transport of *T. gondii* oocysts and incorporation into marine food webs has been addressed in several investigations. In temperate regions, rainfall can mobilize the transport of fecally-deposited oocysts into watersheds that drain into coastal waters (Miller et al., 2002; VanWormer et al., 2016), while in colder locales early snowmelt may also provide a means for mobilizing oocysts deposited the previous year (Simon et al., 2013b). In coastal waters, entry of *T. gondii* oocysts into the marine food chain is facilitated through their accumulation in organic flocs that serve as food for fish and invertebrates (Shapiro et al., 2012; Shapiro et al., 2014). While these cold-blooded animals may not serve as true intermediate hosts, *T. gondii* oocysts can be ingested and retained by fish (Massie et al., 2010), bivalves (Arkush et al., 2003; Lindsay et al., 2004), and marine snails (Krusor et al., 2015; Schott et al., 2016). Ingestion of these invertebrates has been epidemiologically linked to increased risk of *T. gondii* infections in higher trophic animals such as marine mammals (Johnson et al., 2009) or people consuming seafood (Jones et al., 2009).

## 5 Environmental transmission of oocysts

### 5.1 Oocysts in water

Humans and susceptible animal hosts can be exposed to *Toxoplasma gondii* oocysts in the environment through drinking water contaminated with felid feces. Oocysts can survive various inactivation procedures especially those using chemical reagents (Dubey, 2004). Oocysts remain viable in water even after exposure to aqueous 2% sulfuric acid for at least 18 months at 4 °C; they also resist detergents or disinfectant solutions such as sodium hypochlorite. Drinking-water treatment plants using chlorination as the sole method of disinfection could therefore supply water containing infective oocysts.

Interest in detecting *T. gondii* oocysts in the environment is emerging due to recent outbreaks of waterborne toxoplasmosis in humans. Until recently, *Toxoplasma* was not considered to be a significant waterborne pathogen like other protozoan parasites, such as *Cryptosporidium* and *Giardia* which have been linked with numerous waterborne outbreaks, including a very large *Cryptosporidium* outbreak reported in Milwaukee, Wisconsin, USA (MacKenzie et al., 1995). The first *T. gondii* waterborne outbreak was associated with exposure to water from a jungle stream in Panama in 1979, resulting in infection of 39 soldiers who used three water sources for drinking water (Benenson et al., 1982). An epidemiological investigation identified the source as creek water contaminated by oocysts excreted by wild felids. A prominent outbreak was then reported in British Columbia, Canada in 1995, with 100 cases of human acute *Toxoplasma* infection (Bowie et al., 1997). A municipal water system that used unfiltered and chloraminated surface water was the likely source of contamination by cougar and/or domestic cat feces (Aramini et al., 1999). In 1999, drinking water was also reported as the vehicle of infection among Jains, a community of strict vegetarians in India (Hall et al., 1999).

Waterborne toxoplasmosis has been reported most often from Brazil, in both epidemic as well as endemic transmission patterns. The largest outbreak in the published literature, with 290 human cases, was reported in Brazil and involved an unfiltered water reservoir (Keenihan et al., 2002). Moreover, a high *T. gondii* prevalence related to drinking unfiltered water was found in Brazilian communities where endemic toxoplasmosis is prevalent (Bahia-Oliveira et al., 2003). Likewise, a large toxoplasmosis outbreak in Brazil in 2001–2002 was associated with the consumption of contaminated water from a municipal reservoir, which was vulnerable to infiltration due to its precarious management state. This outbreak was thought to be responsible for infection of 155 persons served by an underground tank reservoir delivering unfiltered water (de Moura et al., 2006).

While most *T. gondii* waterborne outbreaks have been described from low-middle income countries, epidemiological investigations have also linked water with exposure to *T. gondii* in people living in high-income regions. In the USA, the NHANES survey data (1999–2004; 2009–2010) demonstrated that among US-born participants, individuals drinking well water, as well as public/private company-provided tap water with no additional at-home water treatment devices, were significantly more likely to be seropositive for *T. gondii* as compared with participants who used home treatment devices (Krueger et al., 2014). Despite limitations in the NHANES cross-sectional survey, the association between water and *T. gondii* infection in the USA warrants further research.

Detection methods for *T. gondii* oocysts in the environment are underdeveloped in comparison to other protozoan parasites (i.e., *Cryptosporidium* spp. and *Giardia duodenalis*). Detection of oocysts has relied on a combination of bioassays, microscopy, and molecular assays as reviewed by Dumetre and Darde (2003) and more recently by Bahia-Oliveira et al. (2017). Although PCR does not demonstrate infectivity, it is widely applied for estimating the presence of water contamination with pathogenic protozoa. Immunofluorescence assays commonly employed to detect other waterborne protozoa, namely *Cryptosporidium* and *Giardia* oo(cysts), are described (Dumetre and Darde, 2005, Dumetre and Darde, 2007), but are not commercially available for *T. gondii* (because of a lack of suitable monoclonal antibodies) and they have not been extensively tested in field conditions. One approach recently described in the literature for capture of *T. gondii* oocysts from concentrated water utilized lectin-coated magnetic beads (Harito et al., 2017), however additional application of this methodology is needed to evaluate feasibility and sensitivity when applied to large volumes of diverse water types.

Similar to detection of other protozoan parasites, an initial concentration step is required due to the relatively dilute distribution of *T. gondii* oocysts in environmental water sources. Concentration methods include membrane filtration, capsule filtration, hollow fiber ultrafiltration, flocculation, and centrifugation. For direct visualization of *T. gondii*, microscopy-based methods were employed based on the autofluorescence nature of *T. gondii* oocyst and sporocyst walls (Lindquist et al., 2003). While cost effective, methods that rely on microscopy-based detection require molecular confirmation to definitively identify oocysts as *T. gondii*, because other apicomplexan parasites have an identical morphology (e.g., *Hammondia*, *Besnoitia*, and *Neospora* oocysts).

Isolation of *T. gondii* from water using mouse bioassays provides definitive identification of viable parasites, but this approach is laborious, costly, and time-consuming. Interestingly, this approach was successfully applied in the 2001 Brazil outbreak, where water collected from the suspected reservoir was filtered and multiple bioassays were performed in cats, chickens and pigs fed with the membrane filters, leading to parasite isolation and genotyping (de Moura et al., 2006). To date, this report represents the sole outbreak in which detection of *T. gondii* in the implicated water source was achieved. Multilocus DNA sequencing has identified a nonarchetypal strain of *T. gondii* as the causal agent of this waterborne outbreak. The strain, isolated from a water supply epidemiologically linked to the outbreak, was virulent to mice, and it has previously been identified as BrI (Vaudaux et al., 2010). Because no parasites were recovered from infected individuals, no direct determination of the responsible strain type(s) could be performed. Moreover, Vaudaux et al. (2010) used a serologic strain-typing assay to determine “serotypes” for a group of such individuals. There was a dominant serotype in the majority (65%) of individuals, which was indistinguishable from the serotype found in serum from mice that had been infected with isolates from the implicated water supply. Furthermore, the outbreak strains were genotyped at multiple polymorphic loci and found to be clonotypic and nonarchetypal. Hence, data support prior evidence that the municipal cistern was the point source of this clonal outbreak.

Due to ethical concerns, cost and the time-consuming nature of bioassays, rapid and sensitive pathogen detection methods are essential for public health and water quality industries. PCR has already been described as more rapid, sensitive, and specific for *Cryptosporidium* detection in environmental water (Fontaine and Guillot, 2003). Therefore, molecular detection has been proposed for sensitive and rapid detection of *T. gondii* oocysts, and assays include both conventional and real-time, quantitative PCR. Villena et al. (2004) have previously compared bioassays and molecular approaches and reported better performance of PCR, with sensitivity varying from <10 to >1000 oocysts/l, depending on water type. Using this PCR method in environmental samples, Aubert and Villena (2009) reported detection of DNA in raw surface water, underground water and public drinking water, while isolation of *T. gondii* by bioassay was unsuccessful (Aubert and Villena, 2009). Additional molecular approaches using loop-mediated isothermal amplification (LAMP) have also been described with better results than nested PCR and applied to *T. gondii* detection in environmental samples (Sotiriadou and Karanis, 2008).

Application of PCR for detection of *T. gondii* in water has been applied in numerous studies worldwide, and recently reviewed by Bahia-Oliveira et al. (2017). In Colombia, the prevalence of *T. gondii* DNA in 46 samples of drinking water was 58.6% (Trivino-Valencia et al., 2016). Similar prevalences were reported in raw and treated water in Bulgaria at 48% (Sotiriadou and Karanis, 2008) and in Poland at 37.5% (Sroka et al., 2006). In comparison, lower prevalences of *Toxoplasma* in water have been reported via real-time PCR in Scotland at 8.7% (*N* = 1411) (Wells et al., 2015), and in France's Champagne-Ardenne region at 7.7% (*N* = 482), where some of the positive samples were obtained from public drinking water (Aubert and Villena, 2009).

It is imperative that any molecular detection technique applied to environmental samples be followed by sequence analysis for definitive confirmation of *T. gondii* DNA due to a potential for non-target amplification by flora and biota commonly present in environmental habitats, even when using primers shown to be specific for *T. gondii* when compared with other coccidian or enteric pathogens (Shapiro et al., 2015). Moreover, as certain molecular approaches do not easily lend themselves to downstream sequence analysis (e.g., qPCR or LAMP), additional conventional PCR may be necessary as an intermediary step. Thus, although multiple methods for oocyst detection are described, the lack of standardized assays for detection of *Toxoplasma* oocysts in the environment has likely contributed to an underestimation of the role that oocysts play in the epidemiology of *T. gondii* in human populations. Additional research on the burden of environmental contamination with *T. gondii* oocysts including different water sources is necessary to accurately estimate the risk of oocyst ingestion by people and animals.

### 5.2 Oocysts in soil

*Toxoplasma gondii* oocysts can contaminate soil after infected felids shed the parasite in their feces (Gilot-Fromont et al., 2012). Because of the limited definitive host species for *T. gondii* (felids only), oocysts are not randomly distributed in soil, but rather tend to concentrate in or near cat defecation sites (Afonso et al., 2007) (Table 1). However, oocysts can be further dispersed within the soil column or to other matrices by wind, earthworms and arthropods, as well as by rain (Dumetre and Darde, 2003). Exposure to soil contaminated with *T. gondii* oocysts is one of the main risk factors for infection in people (Cook et al., 2000; Spalding et al., 2005; Jones et al., 2009; Egorov et al., 2018) and is likely the main route of transmission for domestic food-producing animals and many other intermediate hosts including rodents and birds (Gilot-Fromont et al., 2012). Table 1 summarizes published reports that have focused on detection and prevalence of *T. gondii* in soil. Overall, the reported prevalence of *T. gondii* in soil from various locations ranges from 0% (Hawaii, USA) to nearly 50% (Northeastern France).

**Table 1:** Reports documenting the detection and prevalence of *Toxoplasma gondii* in soil.

Country/region (reference)	Location (urban/rural)	Oocyst recovery method	Detection method (gene target)	Sample size and prevalence	Sequence confirmation	Key findings
France/Lyon (Afonso et al. 2008)	Urban	Sucrose flotation	Real-time PCR (529-bp RE)	*N* = 117 9.4%	NR	*T. gondii* DNA most commonly detected in cat latrines
Poland/Tricity (Lass et al., 2009)	Urban	Sodium nitrate flotation	Conventional PCR (B1)	*N* = 101 17.8%	Yes (two random samples)	Type I and II genotypes detected
China/Hubei Province (Du et al., 2012b)	Urban	NR	Conventional PCR (B1 and 529-bp RE); LAMP (MIC3)	*N* = 252 PCR:16.3% LAMP: 23.02%	Yes (four random samples)	Significantly lower *T. gondii* DNA detected in Fall and Winter
China/Hubei Province (Du et al., 2012a)	Rural	NR	Conventional PCR (B1); LAMP (MIC3)	*N* = 95 PCR: 21.1% LAMP: 37.9%	NR	
France/Northeastern (Gotteland et al., 2014)	Rural	Modified sucrose flotation (Lélu et al., 2011): sugar-water interface used	Real-time PCR (529-bp RE)	*N* = 243 29.2%	NR	*T. gondii* DNA was not restricted to areas of high cat density
China/Nanjing Region (Liu et al., 2017)	Rural	NR	Conventional PCR (ITS1)	*N* = 700 1%	Yes (all)	*T. gondii* only detected during Fall (3.3%) and Winter (0.56%)
China/Gansu province (Wu et al., 2017)	Urban	Suspension in distilled water	Nested PCR (B1); LF-RPA (B1)	*N* = 35 Nested PCR: 14.3% LF-RPA: 14.3%	Yes (all)	
France/Northeastern (Simon et al., 2017)	Rural	Modified sucrose flotation (Lélu et al., 2011): entire supernatant used	Real-time PCR (529-bp RE)	*N* = 558 49.82%	NR	*T. gondii* detected near core cat colony areas; and associated with latrines and scattered feces, as compared with random soil samples
USA/O'ahu (Davis et al., 2018)	Urban and rural	Modified sucrose flotation method (Lélu et al., 2011)	Conventional PCR (GRA6)	*N* = 120 0%	NR	*T. gondii* not detected in soil samples.
USA/California (de Wit et al., *in preparation* 2019)	Urban	Modified sucrose flotation method (Lélu et al., 2011)	Nested PCR (ITS-1)	N = 482 5.6%	Yes (all)	*T. gondii* detection associated with wet season, coastal sites, and small cat colony size.

NR = not reported; PCR = polymerase chain reaction; LAMP = loop-mediated isothermal amplification; LF-RPA = lateral flow recombinase polymerase amplification.

Oocyst viability and persistence in soil can be influenced by environmental factors such as humidity, temperature, vegetation and soil characteristics. Oocysts lose their ability to sporulate when exposed to freezing conditions (−21 °C for 1 day or − 6 °C for 7 days), extreme heat (50 °C for 10 min) or extreme solar radiation. However, once sporulated, oocysts are highly resistant and can persist in moist soil for up to 18 months when exposed to temperatures ranging from −20 °C to 35 °C (Dumetre and Darde, 2003). For example, the prevalence of *T. gondii* in soil tends to be higher in wet or moist seasons characterized by mild temperatures (Du et al., 2012b) (Table 1). In addition, soil with high concentrations of clay and sand can interfere with molecular methods of detection given their high content in organic and abrasive particles, respectively; however, whether these characteristics also affect retention and viability of oocysts in the soil column is unknown.

Despite the known risk of exposure to *T. gondii* oocysts through contact with soil (e.g., gardening or playing in sandboxes), confirming the presence and estimating the load of *T. gondii* in soil has proven difficult due to limitations in currently available methods of detection (Bahia-Oliveira et al., 2017). Given the likely heterogeneous dispersion of oocysts in soil, accurate estimations of *T. gondii* prevalence and load require large sample sizes, many of which may contain small quantities of oocysts, as well as oocysts of different ages, which may hinder the sensitivity of current detection methods (Lélu et al., 2011; Robert-Gangneux & Dardé, 2012). Commonly used methods for oocyst detection in soil include microscopy and molecular assays (Bahia-Oliveira et al., 2017; Dumetre and Darde, 2003). For both methodological approaches, initial concentration and purification of oocysts from soil is required. To accomplish these steps, most studies have applied flotation techniques using sucrose (specific gravity ≥ 1.15), cesium chloride, or sodium nitrate saturated solutions (Dumetre and Darde, 2003; Lass et al., 2009; Lélu et al., 2011) (Table 1). Molecular techniques for detection of *T. gondii* DNA in soil include conventional PCR, nested PCR, real-time quantitative PCR, loop-mediated isothermal amplification (LAMP), and to a lesser extent, lateral flow recombinase polymerase amplification (LF-RPA) (Table 1). Spiking experiments using various numbers of oocysts aliquoted into soil samples are commonly used to validate the sensitivity and limits of detection of molecular assays (e.g., (Lélu et al., 2011; Liu et al., 2017; Wu et al., 2017). Molecular assays are currently the most sensitive and efficient methods for detecting *T. gondii* in soil; nevertheless, it is important to confirm amplification results through sequence analysis (Bahia-Oliveira et al., 2017)

An intriguing route of *T. gondii* oocyst transmission that is not often discussed is the role of mechanical vectors in disseminating oocysts to true intermediate hosts in terrestrial habitats. For example, invertebrates including cockroaches (Wallace, 1973), earthworms, and flies (Frenkel et al., 1975) have been shown to act as paratenic or mechanical hosts through which oocysts can pass inertly. In addition, dogs have been found to defecate *T. gondii* oocysts in their feces, presumably from passive gastrointestinal transport of oocysts that they ingest through coprophagy (feeding on cat feces) (Lindsay et al., 1997); mechanical transmission of oocysts on dog fur following rolling in cat feces has also been proposed as a mechanism for domestic dogs to disseminate oocysts, especially to children (Frenkel et al., 2003).

## 6 Foodborne transmission of oocysts

### 6.1 Oocysts on fresh produce

For decades, foodborne transmission of *Toxoplasma gondii* has traditionally referred to the ingestion of tissue cysts in raw or poorly cooked meats (Guo et al., 2015). However, it has become increasingly evident that ingestion of oocysts on fresh produce and other foods is under recognized, and the significance of this route of transmission to humans is not entirely clear. Unlike other foodborne protozoan parasites, which have been implicated in numerous illness outbreaks worldwide, there have been only two reported outbreaks of toxoplasmosis associated with the consumption of fresh produce or juice. Ekman et al. (2012) described an outbreak of acute toxoplasmosis at an industrial plant in Brazil, which was associated with the consumption of green vegetables. In a second outbreak in Brazil, Morais et al. (2016) identified 73 cases of acute toxoplasmosis which were associated with the consumption of açaí juice (Morais et al., 2016). Robertson (2016) concluded that the scarcity of reported toxoplasmosis cases associated with the consumption of salad vegetables might be due to the many potential routes of infection for this parasite, and the fact that infections are often asymptomatic.

While numerous surveillance studies have been performed worldwide on the presence of foodborne parasites (e.g., *Giardia*, *Cryptosporidium*, *Cyclospora*, and others) on fresh produce (Dixon, 2015), fewer such studies have been reported on *T. gondii*. In addition to the under recognition of this mode of transmission for *T. gondii*, a major reason for the paucity of surveillance studies on fresh produce is the lack of standard detection methods for this parasite. In the past decade, there have only been several reports on the occurrence of *T. gondii* on fresh produce, based on different elution methods and a variety of microscopy- and molecular-based assays (Table 2). An additional approach utilizing LAMP was recently validated for detection of *T. gondii* on salads (Lalle et al., 2018), but this method has not been applied in surveillance investigations to date. The application of recently developed methods that can discriminate oocyst viability is essential for further discerning whether detected *T. gondii* DNA on produce is derived from infectious versus non-viable parasites (Hohweyer et al., 2016; Travaille et al., 2016).

**Table 2:** Reports documenting the presence of *Toxoplasma gondii* on fresh produce.

Country (reference)	Produce type	Oocyst recovery method	Detection method (gene target)	Sample size and prevalence	Sequence confirmation	Molecular characterization (method)
Saudi Arabia (Al-Megrin, 2010)	Leafy vegetables	Wash, passive	Microscopy	*N* = 470 6.6%	No	
Poland (Lass et al., 2012)	Radish, carrots, lettuce	Flocculation	qPCR (B1, nested PCR SAG2)	*N* = 216 9.7%	No	Types I and II (RFLP)
Pakistan (Shafa-ul-Haq et al., 2014)	Market vegetables	Wash – sedimentation or flotation	Microscopy	*N* = 500 1.9%	No	
Canada (Lalonde and Gajadhar, 2016)	Retail leafy greens	Wash, orbital shaking	qPCR - melting curve analysis (18S rDNA)	*N* = 1171 0.26%	Yes (98–99% identity)	
Brazil (Marchioro et al., 2016)	Leafy greens	Wash, manual	PCR (B1, 529 bp RE)	*N* = 238 3.8%	No	
Italy (Caradonna et al., 2017)	RTE salads	Wash, orbital shaking	Microscopy; qPCR - melting curve analysis (B1)	*N* = 648 0.8%	Yes	Type I (Sequencing)
Brazil (Ferreira et al., 2018)	Organic leafy greens	Wash, manual	PCR (529 bp RE)	*N* = 83 9.5%	Attempted, not successful	

Contamination of fresh produce with *T. gondii* oocysts may occur at primary production sites on farms (e.g., cultivation in soil contaminated with cat feces) or by means of contact with fecally-contaminated water used in irrigation, washing or processing (Fig. 2). These direct and indirect sources of contamination are particularly prevalent in low- and middle-income countries where hygiene, sanitation and water quality may be sub-optimal (see Section 3.1; (Dixon, 2016)). Largely as a result of the global food trade, particularly the importation of exotic and out-of-season fruits and vegetables, risk of exposure to *T. gondii*-contaminated produce is also present in high-income countries. With the increasing domestic cat population in many regions worldwide, there will consequently be more *T. gondii* oocysts released into the environment resulting in a greater risk of food and waterborne transmission to humans and other animals (Bahia-Oliveira et al., 2017).

As fresh produce is often consumed raw, effective control measures to minimize parasite contamination, inactivate or reduce parasite viability, or physically remove oocysts, are imperative to reduce the risk of *T. gondii* exposure to consumers. At the preharvest level, control measures to reduce the likelihood of contamination of produce with *T. gondii* include mainly the use of treated water for irrigation, and restricted access to gardens and croplands by cats. However, once crops are contaminated, the resistance of *T. gondii* oocysts to environmental stressors should be taken into consideration with respect to their survival. *T. gondii* oocysts are very robust and likely persist on produce for weeks to months (Robertson, 2016; Bahia-Oliveira et al., 2017). Kniel et al. (2002) demonstrated that sporulated oocysts could attach to and remain infectious on berries for at least 8 weeks under refrigeration. Although there are little supporting data, desiccation may be responsible for significant inactivation of parasite stages on fresh produce in the field or during storage, and on surfaces and equipment.

Postharvest control measures include primarily the use of treated water for washing and processing produce, and for cleaning equipment. The use of chemical and physical disinfectants, either directly on foods, or on surfaces and equipment, represent other potential barriers to the foodborne transmission of parasites (Erickson and Ortega, 2006). As discussed in previous sections of this review, *T. gondii* oocysts are very resistant to chemical treatments and sanitizing regimens used in the water and food industries. In terms of physical disinfection, a variety of technologies have been shown to be effective in the destruction of parasites on fresh produce. For example, gamma irradiation of protozoa has been shown to be an effective means of decontaminating fresh fruits and vegetables. Dubey et al. (1998) reported that sporulated *T. gondii* oocysts inoculated onto raspberries were inactivated at 0.4 kilogray (kGy) and concluded that 0.5 kGy would be effective in killing coccidian oocysts on fruits and vegetables. More recently, Lacombe et al. (2017) (Lacombe et al., 2017) demonstrated that the viability of *T. gondii* oocysts could be significantly reduced following irradiation treatment of just 0.2 kGy. High hydrostatic pressure (or high-pressure processing, HPP) has also shown some promise in the inactivation of protozoan parasites on fresh produce and in juices. Lindsay et al. (2008) reported that *T. gondii* oocysts inoculated onto raspberries were rendered noninfectious to mice when the berries were exposed to 340 MPa for 60 s in a commercial HPP unit. Although studies have been performed on the efficacy of UV treatment in the inactivation of *T. gondii* oocysts in water, results have been inconsistent (Bahia-Oliveira et al., 2017).

At the consumer level, peeling of fruits and vegetables whenever possible will also reduce the risk. As with other foods, the adherence to safe food-handling practices, such as hand washing and the use of separate cutting boards, knives and other utensils will help to reduce the likelihood of cross-contamination of fresh produce. Treatments such as cooking and freezing may be used as final barriers against transmission of *T. gondii* but, since fresh produce is very often consumed raw, these methods are not always relevant. Household freezing should not be recommended as the sole means of inactivating parasites in foods because sporulated *T. gondii* oocysts have been shown to tolerate temperatures as low as −20 °C for up to 28 days (Frenkel and Dubey, 1973).

### 6.2 Oocysts in seafood

Compared with meat-producing and poultry animals, research on seafood species contaminated with *Toxoplasma gondii* represents a relatively new field of study. For example, while literature on cystic *T. gondii* in food animals can be dated as far back as the 1960s (Jacobs et al., 1960), the first published study reporting natural *T. gondii* contamination in shellfish was published in 2008 where the atypical Type X genotype was detected in a single mussel from central California, USA (Miller et al., 2008). Between 2008 and 2015, eight additional studies describing the presence of *T. gondii* in diverse seafood species were reviewed by Bahia-Oliveira et al. (2017). Overall, *T. gondii* contamination has been detected in wild and commercial seafoods such as clams, mussels, oysters, and fishes from several countries including Brazil, China, Turkey, and the USA (Miller et al., 2008; Esmerini et al., 2010; Putignani et al., 2011; Aksoy et al., 2014; Zhang et al., 2014; Shapiro et al., 2015; Staggs et al., 2015). Since 2015, two additional studies reported the presence of *T. gondii* in supermarket-purchased mussels in New Zealand (Coupe et al., 2018), and in wild clams from Tunisia (Ghozzi et al., 2017).

Methods for detecting *T. gondii* in seafoods remain inconsistent across studies, which hinders direct comparison of prevalence and distribution among studies. Common approaches utilize whole tissue homogenates, gills, digestive tissues, or hemolymph (from shellfish) as the testing matrix and apply different molecular assays based on conventional or real-time PCR. The ability of shellfish to bioaccumulate oocysts from contaminated waters renders these species as efficient biosentinels for *T. gondii* contamination of aquatic habitats, both in marine (Arkush et al., 2003; Lindsay et al., 2004) as well as freshwater habitats (Palos Ladeiro et al., 2014). The presence of *T. gondii* in shellfish and fish is not only a risk to susceptible marine wildlife (as discussed in Section 4.2), but also to people. Jones et al. (2009) (Jones et al., 2009) reported that eating raw shellfish is a significant risk of *T. gondii* infection in humans. With anthropogenic influences and climate variability scenarios that forecast increased pathogen contamination of aquatic ecosystems (VanWormer et al., 2016), the extent of *T. gondii* presence and load is likely to rise in seafood species consumed by wildlife or people. Method standardization focusing on affordable, rapid and accurate assays for *T. gondii* detection is imperative, as well as application of molecular approaches for distinguishing presence of viable from non-viable oocysts. Ultimately, the most sustainable approach for reducing the risk of *T. gondii* exposure through consumption of seafood should focus on reducing *T. gondii* contamination at its source (e.g., domestic cat management strategies), as well as mitigating the flow of contaminated runoff to water bodies.

## 7 Managing oocyst-borne infections

Reducing the risk of oocyst-borne *Toxoplasma gondii* infections in animals and people should target three distinct but not mutually exclusive factors: 1) reducing felid contributions of oocysts into the environment; 2) preventing oocyst contamination of water, soil, and foods; and 3) physically removing or inactivating oocysts in water and foods such as shellfish and produce. Detailed recommendations to achieve these steps have been further outlined by Bahia Oliveira et al., (2018), and are summarized below:

• Domestic cats should be kept indoors, and efforts made to reduce the likelihood of intermediate hosts entering the household (e.g., rodents, etc.).
• Domestic cats should be neutered or spayed to help reduce stray cat populations.
• Domestic cats should be fed by a specially formulated diet (canned or dry food), not human food.
• Feces from pet cat litter boxes should be collected daily, sealed in a bag, and disposed of in the garbage (not flushed in the toilet). Litter box must be cleaned with hot water (to avoid oocysts dissemination).
• Sand boxes and play areas should be covered to restrict cat defecation in the area.
• Private and community gardens should likewise restrict access to cats, via fencing or hazing tools such as water sprinklers.
• Gloves should be worn when gardening, and hands thoroughly washed afterwards.
• Filtered or bottled water should be consumed if living or travelling in an endemic region.
• Persons at-risk (e.g., pregnant women and immunocompromised individuals seronegative for toxoplasmosis) should avoid recreating in fresh or marine waters in endemic regions, or in non-endemic regions if in close proximity to overland runoff from heavily populated zones.
• Seafood should be thoroughly cooked to inactivate oocysts. Heating oocysts at 80 °C for 2 min has been shown to render them nonviable (Travaille et al., 2016).
• Produce should be washed with drinking water (or with filtered or bottled water if living or travelling in an endemic region); peeling fruits and vegetables for at-risk persons is also recommended.
• Common household products such as detergents, antimicrobial soaps, and bleach are *not* effective at killing oocysts, and their use for this purpose is not recommended.
• Municipal and ecosystem-level management strategies should be implemented to reduce the overall flux of oocysts mobilized to nearshore waters through runoff. Specific recommendations include wetland preservation and restoration (Shapiro et al., 2010), replacement of impermeable surfaces such as asphalt with alternative permeable paving options (Newman et al., 2013), and storm-water treatment processes including bioswales and raingardens (Virahsawmy et al., 2014).

## 8 Future risk and opportunities for research

Future projections of human population growth coupled with climate variability scenarios will likely lead to greater likelihood of environmental contamination with *Toxoplasma gondii* oocysts (Shapiro, 2012). Domestic cat density is associated with human density (Liberg et al., 2000), thus more people often mean more pets, and in this case, more definitive hosts for *T. gondii*. Concurrently, projections of weather patterns in many regions of the world forecast increase in intense rainfall events interspersed with longer periods of drought (Heim, 2015). Given the robust nature of *T. gondii* oocysts and their ability to persist over long durations in feces and soil, a greater force of intense rainfall events will lead to enhanced mobilization of oocysts that have accumulated during dry periods. The risk of oocyst-borne infections for humans and animals may, therefore, rise in coming decades. For this reason, it is imperative that collaborative, multidisciplinary expert teams from around the world tackle applied research on the biology, detection, epidemiology and ecology of *T. gondii* oocysts in the environment. Specific opportunities for future research are outlined below:

• The current lack in standardized *T. gondii* oocyst detection and quantification methods hinders effective establishment of surveillance programs for monitoring oocyst presence in water, soil and foods. This also prevents accurate comparison of *T. gondii* contamination across studies that often use different approaches for oocyst purification, nucleic acid extraction and amplification, as well as sequence analysis and confirmation. Establishing a consensus among laboratories and regulatory agencies regarding optimized protocols for oocyst detection should facilitate enhanced surveillance for the parasite. Early and accurate detection of *T. gondii* in water and foods is essential for reducing the occurrence of toxoplasmosis outbreaks in endemic geographical regions.
• In addition to recommended protocols for *T. gondii* DNA detection, it is imperative that methods for distinguishing oocyst viability are continuously improved and increasingly applied in investigations on *T. gondii* prevalence in the environment. While oocysts are remarkably persistent, some decay over time under diverse environmental conditions must certainly occur (Lélu et al., 2012). Thus, viability discrimination assays are essential for accurately characterizing the risk that *T. gondii* poses to exposed populations when its DNA is identified in water, soil or foods (Rousseau et al., 2019).
• Innovative and reliable inactivation methods are needed for application on contaminated water and foods for removing *T. gondii* oocysts or reducing their infectivity. Filtration systems (sand filtration, ultra- and nano-filtration) appear to be the most effective methods for removing oocysts from drinking water prior to its chemical disinfection and distribution to consumers. Disinfection of fresh produce is more problematic as treatments have to kill oocysts without altering the organoleptic properties of food. In this regard, pulsed light is an attractive technology for inactivating bacterial spores and *C. parvum* oocysts on berries (Le Goff et al., 2015; Rousseau et al., 2018), however this approach requires experimental validation for *T. gondii*.
• Investigations are warranted to explore the frequency of oocyst re-shedding in nature, particularly in feral domestic cats and wild felids that are exposed repeatedly and to diverse genotypes of the parasite. Oocyst shedding data (prevalence and number of oocysts shed) for more populations of free-ranging domestic and wild felids will enhance predictions of *T. gondii* load and distribution in the environment. An enhanced understanding of when, where and how many oocysts are present in soil will help target intervention measures for reducing the risk of exposure to oocysts through recreation (e.g. children playing in sand boxes), gardening, or ingestion of contaminated fresh produce.
• Transport and fate questions remain regarding the ability of *T. gondii* to disseminate in the environment, and a particularly puzzling finding is the presence of *T. gondii* in deep ocean dwelling mammals or in remote regions devoid of cats, such as Antarctica. Mechanisms that allow for oocyst movement via ocean currents or within mechanical hosts such as fish should be further explored. In addition, the effect of habitat change and climate variability on oocyst transport and fate warrant further research. Insight on oocyst transport can reveal how food resources including marine mammals, harvested for sustenance by indigenous populations (Sharma et al., 2018), or shellfish become contaminated – which is key for managing *T. gondii* infections in the people consuming them.
• Safe and efficacious vaccines to protect livestock against oocyst-borne *T. gondii* infections are needed. The currently available TOXOVax® is used in some countries (e.g., New Zealand and the UK) but prohibited in others (USA and Canada) due to its modified-live nature and subsequent risk of accidental zoonotic transmission. *T. gondii* is a leading cause of abortions in sheep and goats in Canada (Hazlett et al., 2013), and a vaccine is urgently needed to reduce economic losses, improve animal welfare, and reduce the burden of infected meat or contaminated dairy products that are sold to consumers.
• Finally, it is noteworthy to conclude that regions where *T. gondii* is most prevalent, and subsequently people and animals suffer greatest morbidity and mortality due to this parasite, are also low- and middle-income countries, where resources for research are limited, and the need for improving water and food sanitation are greatest. It is imperative, therefore, that international collaborations form to support high caliber investigations in regions that need it most, and that dissemination of expertise and resources for *T. gondii* research among countries is supported.

## Conflict of interest

The authors report no conflict of interest.
